# Primary results of a randomized two‐by‐two factorial phase II trial comparing neoadjuvant chemotherapy with two and four courses of cisplatin/S‐1 and docetaxel/cisplatin/S‐1 as neoadjuvant chemotherapy for advanced gastric cancer

**DOI:** 10.1002/ags3.12352

**Published:** 2020-07-16

**Authors:** Tsutomu Hayashi, Takaki Yoshikawa, Kentaro Sakamaki, Kazuhiro Nishikawa, Kazumasa Fujitani, Kazuaki Tanabe, Kazunari Misawa, Takanori Matsui, Akira Miki, Hiroshi Nemoto, Tetsu Fukunaga, Yutaka Kimura, Jun Hihara

**Affiliations:** ^1^ Gastric Surgery National Cancer Center Hospital Chuo‐ku Japan; ^2^ Center for Data Science Yokohama City University Yokohama Japan; ^3^ National Hospital Organization Osaka National Hospital Osaka Japan; ^4^ Osaka General Medical Center Osaka Japan; ^5^ Graduate School Hiroshima University Hiroshima Japan; ^6^ Aichi Cancer Center Hospital Nagoya Japan; ^7^ Aichi Cancer Center Aichi Hospital Nagoya Japan; ^8^ Kobe City Medical Center General Hospital Kobe Japan; ^9^ Fujigaoka Hospital Showa University Yokohama Japan; ^10^ University Hospital St. Marianna University School of Medicine Kawasaki City Japan; ^11^ Sakai City Medical Center Sakai Japan; ^12^ Hiroshima City Asa Hospital Hiroshima Japan

**Keywords:** cisplatin/S‐1, docetaxel/cisplatin/S‐1, gastric cancer, neoadjuvant therapy

## Abstract

**Aim:**

Neoadjuvant chemotherapy (NAC) is promising to improve the survival of resectable gastric cancer. However, suitable regimen and treatment duration for NAC have not yet been established.

**Methods:**

We conducted a randomized phase II trial to compare two and four courses of neoadjuvant S‐1/cisplatin (SC) and S‐1/cisplatin/docetaxel(DCS) using a two‐by‐two factorial design for locally resectable advanced gastric cancer. Patients with M0 and either T4 or T3 in case of junctional cancer or scirrhous‐type cancer received two or four courses of SC or DCS. Then, patients underwent D2 gastrectomy and adjuvant S‐1 chemotherapy for 1 year. The primary endpoint was 3‐year overall survival. The planned sample size was 120 eligible patients.

**Results:**

Between October 2011 and September 2014, 132 patients were assigned to CS (n = 66; 33 in 2‐courses and 33 in 4‐courses) and DCS (n = 66; 33 in 2‐courses and 33 in 4‐courses). The 3‐year OS was 58.1% in CS and 60.0% in DCS with hazard ratio of 0.80 (95% CI, 0.48‐1.34), while it was 53.1% in the two courses and 65.0% in the four courses with hazard ratio of 0.72 (95% CI, 0.43‐1.22). In the survival analysis by duration in each regimen, the 3‐year OS was 58.1% for both two and four courses in CS, while it was 48.5% for two courses of DCS and 71.9% for four courses of DCS.

**Conclusions:**

Considering high 3‐year OS, four courses DCS has a value to be tested in a future phase III study to confirm superiority of neoadjuvant chemotherapy for locally advanced gastric cancer.

## Graphical abstract

Overall survival curves stratified by regimens, number of courses.

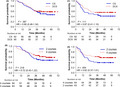

## 1 INTRODUCTION

Gastric cancer is the fifth most common malignancy in the world and the third leading cause of cancer death worldwide.^1^ The complete tumor removal is essential for the cure of gastric cancer. For locally advanced disease, standard treatment is a combination of D2 gastrectomy for the local control^2^ and adjuvant therapy to eradicate micro‐metastasis. However, adjuvant therapy differs depending on region: postoperative chemotherapy with S‐1,^3^ S‐1 plus docetaxel,^4^ or capecitabine plus oxaliplatin in Asia^5^; pre‐ and post‐ operative chemotherapy in the West^6^, ^7^; and postoperative chemoradiation in the US.^8^

In Japan, prognosis for locally advanced disease is still unsatisfactory. More than half of the patients recurred even after D2 and postoperative S‐1 plus docetaxel.^4^ Considering intensity of chemotherapy, preoperative chemotherapy, or so‐called neoadjuvant chemotherapy (NAC), has advantages compared with postoperative chemotherapy.^9^ In Japan, the doublet regimen using S‐1 plus cisplatin (CS) has tested as a neoadjuvant setting in several phase II studies,^10^, ^11^, ^12^ showed safety and efficacy. Moreover, the phase III study testing S‐1 plus Oxaliplatin (SOX) as a NAC setting for locally advanced gastric cancer is ongoing.^13^ Additionally, a triplet regimen added docetaxel to cisplatin and S‐1 (DCS) demonstrated a high response rate for metastatic gastric cancer in phase II trials,^14^, ^15^ indicating that the DCS is a promising regimen for NAC setting. In Europe, perioperative chemotherapy using a triplet regimen, adding docetaxel to fluorouracil and oxaliplatin (FLOT), demonstrated superior efficacy in phase III trial.^7^

In addition to the development of regimen, duration of chemotherapy must be another key for NAC. A certain duration is required for reduction of the tumor cell; however, longer duration may miss the curative operation due to progression of the tumor. Previously, we demonstrated that four courses of CS did not show the survival benefit that two courses of CS did in phase II;^12^ however, the sample size was only 60 patients and inconclusive. Moreover, it remains unclear whether the case for DCS is different from CS.

Based on these, we conducted a randomized phase II trial, COMPASS‐D, to compare NAC using two and four courses of CS and DCS with a two‐by‐two factorial design for locally advanced gastric cancer. The primary endpoint was 3‐year overall survival (OS). In our previous report analyzing the early outcome,^16^ the pathological response rate was found to be similar, regardless of the regimen or course, and the chemotherapy‐related toxicities and surgical outcome of both the regimen and course were found to be feasible. This report clarified the survival results of the COMPASS‐D phase II study, which is intended to select a better regimen and course for the next phase III trial.

## 2 METHODS

The study enrolled patients diagnosed as clinical T4 disease or clinical T3 disease in cases of tumors invading the esophagus and/or of the scirrhous type, including giant type 3 with a maximum diameter of >8 cm. The T and N stage were precisely determined by the protocol. Details of the entry criteria are shown in Table 1.

**TABLE 1:** Entry criteria

Eligibility criteria
Histologically proven adenocarcinoma of the stomach
Clinical T4, or T3 disease in cases of tumors invading the esophagus and/or of the scirrhous type including giant type 3 with a maximum diameter of >8 cm
No pleural effusion, no ascites exceeding the pelvis and no metastasis to the peritoneum, liver or other distant organs, as confirmed by abdominal‐pelvic CT
No metastasis to the lung, mediastinal lymph nodes or other distant organs, as Confirmed by thoracic CT for tumors invading the esophagus
No clinically apparent distant metastasis
Age ranging between 20 and 80 y
ECOG performance status (PS) 0‐1
Sufficient oral intake
No previous treatment with chemotherapy or radiation therapy for any tumors
No previous surgery for the present disease except bypass surgery
Exclusion criteria
Remnant stomach cancer
Synchronous or metachronous cancer (synchronous multiple cancers in the stomach included)
Females with an ongoing pregnancy or breastfeeding, or who were contemplating becoming pregnant
Mental disorders that might affect the ability or willingness to provide informed consent or abide by the study protocol
Systemic treatment with a corticosteroid
Systemic treatment with flucytosine, phenytoin or warfarin potassium
Allergic reaction to iodine
Hypersensitivity to docetaxel, cisplatin or polysorbate
Peripheral neuropathy
Edema
Pneumonitis, lung fibrosis or emphysema in need for oxygen therapy
Active inflammation due to bacteria or fungi
Unstable angina or cardiac infarction within the previous 6 mo
Positivity for HBs antigen or HCV antibody
Unstable hypertension
Diabetes mellitus under treatment.

The patients enrolled in this study received two or four courses of CS or DCS regimens as a neoadjuvant chemotherapy as following: Arm A, two courses of CS; Arm B, four courses of CS; Arm C, two courses of DCS; and Arm D, four courses of DCS. In the CS regimen, S‐1 was given 80 mg/m^2^ orally (p.o.) daily for 3 weeks of a 4‐week cycle, and cisplatin was given as an intravenous infusion of 60 mg/m^2^ on day 8 of each cycle. In the DCS regimen, S‐1 was given 80 mg/m^2^ p.o. daily for 2 weeks of a 4‐week cycle, and 60 mg/m^2^ cisplatin and 40 mg/m^2^ docetaxel were given as an intravenous infusion on day 1 of each cycle. The details of the neoadjuvant chemotherapy treatments have been reported previously.^15^ Following neoadjuvant chemotherapy, patients underwent gastrectomy plus standard D2 lymphadenectomy. D3 lymphadenectomy or combined resection of adjacent organs or minimum resection of the peritoneum is allowed for curative intent, but more extended radical surgery, for instance pancreaticoduodenectomy or Appleby's surgery, are not. After a macroscopic curative resection was achieved, postoperative chemotherapy using S‐1 of 80 mg/m^2^ p.o. daily for 28 days, every 6 weeks, is initiated within 6 weeks after surgery and continued for 1 year.

After surgery, the patients received a physical examination, laboratory test, and abdominal computed tomography scan at least once every 6 months until 3 years after the accrual. Recurrence was confirmed by imaging studies, including computed tomography, ultrasonography, laparoscopy, gastrointestinal radiography, and endoscopy. Individual patients were followed up for at least 5 years after the accrual in accordance with the protocol.

The present study was open‐label, randomized phase II trial of the selection design proposed by Simon et al. This study was designed to evaluate the effectiveness of four courses of NAC compared with two courses, and the effectiveness of the DCS regimen compared with CS. Primary endpoint was 3‐year OS and the key secondary endpoint was progression‐free survival (PFS). The regimen showing a higher 3‐year OS rate was considered more promising for a following phase III trial. Initially, we predicted that the 3‐year OS rate of reference arm would be around 50% as previously reported.^10^ The DCS and four‐course regimens were expected to be 10% better in the 3‐year OS rate than the CS and two‐course regimens. Thus, we assumed that the 3‐year OS rate of DCS and four‐course regimens would be 60%. The sample size required to ensure 85% probability of the correct selection of a more effective regimen was calculated to be 110 patients, with 55 patients per arm. Considering the likelihood of dropouts and ineligible patients, the number of patients to be accrued was set at 120 in total. The primary analysis in this study aimed to estimate the 3‐year OS.

OS and PFS were summarized by the Kaplan‐Meier method in a full analysis set including the randomized patients with start of allocated treatments. The survival curves were compared by the log‐rank test and hazard ratios (HRs) were estimated by the Cox regression models. Subgroup analysis were carried out to assess whether the relative effect from regimens and duration of NAC varies according to baseline characteristics, and treatment effect in each subgroup was presented using a forest plot. All clinical data were held centrally at the data center and analyzed using SAS version 9.4 (SAS Institute Inc).

Eligible patients were registered and subsequently randomised using a centralised dynamic randomization method with the following stratification factors: scirrhous type including giant type 3 (yes/no), tumors invading the esophagus (yes/no), cT3‐4a/T4b disease, lymph node metastasis (yes/no), and institution.

The protocol was approved by the institutional review boards/ethics committees of each participating institution. This trial was registered at the University Hospital Medical Information Network (UMIN) center (ID: UMIN000006387).

## 3 RESULTS

### 3.1 Patients

**FIGURE 1:**
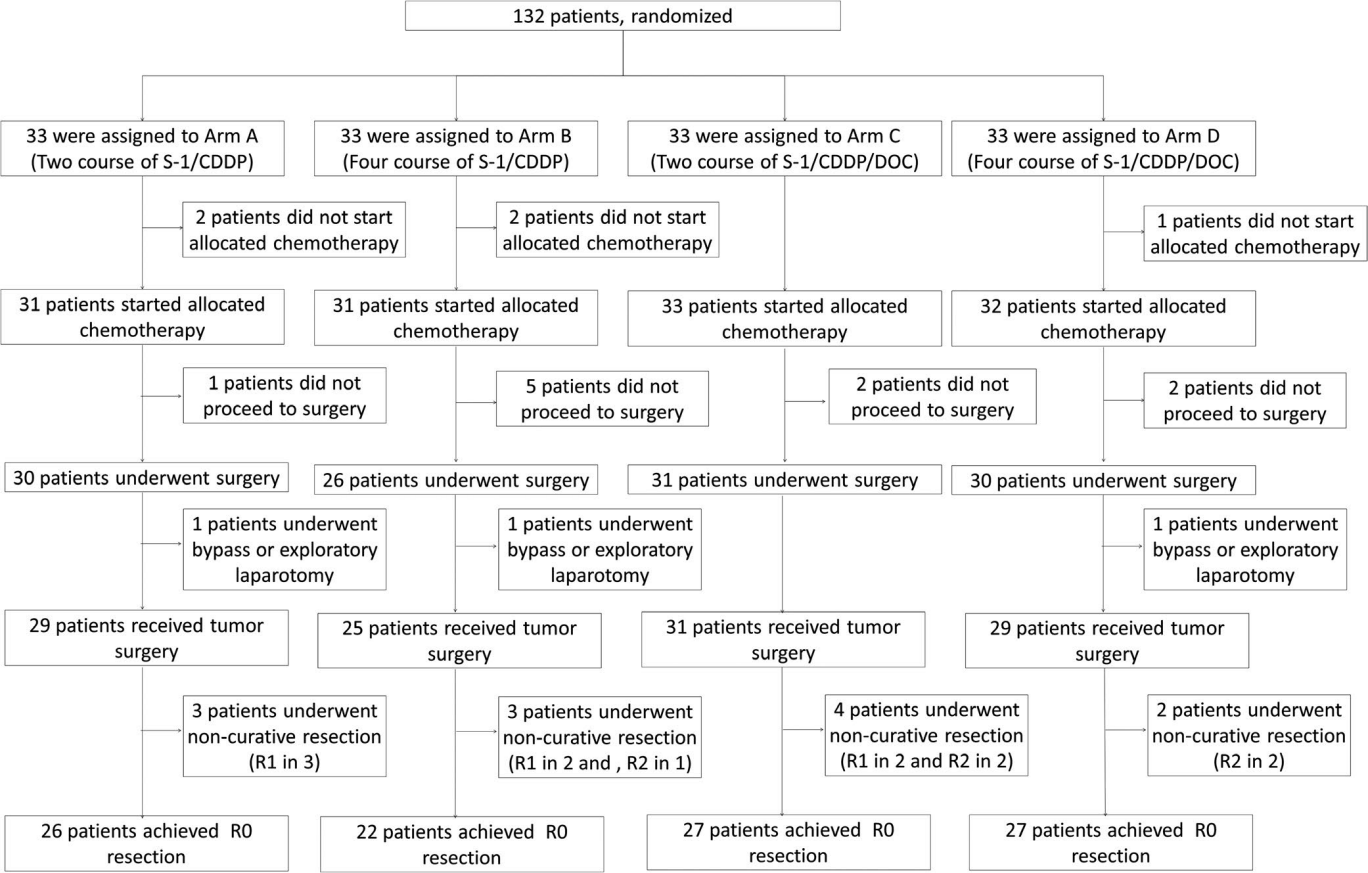
Consort diagram of the present study

Between October 2011 and September 2014, a total of 132 patients were assigned to receive two courses of CS (arm A, n = 33), four courses of CS (arm B, n = 33), two courses of DCS (arm C, n = 33), and four courses of DCS (arm D, n = 33). The patients’ demographics and tumor characteristics of each arm are shown in Table 2. Baseline characteristics were well balanced between the four arms. A consort diagram of patients is presented in Figure 1. The rate of completion of NAC was 77.4% (24 of 31) in arm A, 64.5% (20 of 31) in arm B, 97.0% (32 of 33) in arm C, and 78.1% (25 of 32) in arm D. The rate of completion of neoadjuvant chemotherapy was 71.0% (44 of 62) in the CS arm compared with 87.7% (57 of 65) in the DCS arm, and 87.5% (56 of 64) in the two‐course arm compared with 71.4% (45 of 63) in the four‐course arm. A total of 10 patients did not proceed to surgery because of disease progression. Among the patients who proceeded to surgery, three received a bypass operation because of peritoneal metastasis. Five patients underwent palliative D1 gastrectomy due to stenosis or bleeding by the primary tumor but ultimately received R2 resection because of peritoneal metastasis. All patients without peritoneal metastasis and positive peritoneal cytology received D2 gastrectomy. The R0 resection rate was 83.9% (26 of 31) in arm A, 71.0% (22 of 31) in arm B, 81.8% (27 of 33) in arm C, and 84.4% (27 of 32) in arm D. The R0 resection rate was 77.4% (48 of 62) in the CS arm and 83.1% (54 of 65) in the DCS arm, and 82.8% (53 of 64) in the patients treated with two courses and 77.8% (49 of 63) in the patients treated with four courses. The toxicities related chemotherapy, surgical findings, surgical morbidities, and pathological responses were reported previously.^16^ Briefly, the toxicities of both regimens were acceptable regardless of whether they received two or four courses of treatment. A severe postoperative complication which required reoperation and/or intensive care was not observed in each arm, and surgical morbidities between regimens and courses were similar. No treatment related deaths and surgical mortalities were observed. The pathological response rate defined by less than 10% of the residual tumor remaining was 19.4% in arm A, 19.4% in arm B, 12.1% in arm C, and 18.8% in arm D. Stratifying by the regimen and the duration, that was 19.4% in the CS group and 15.4% in the DCS group and 15.6% in the two‐course group and 19.3% in the four‐course group.

**TABLE 2:** Patients’ characteristics

Characteristics	Variable	Arm A: CS 2 course (N = 31)	Arm B: CS 4 course (N = 31)	Arm C: DCS 2 course (N = 33)	Arm D: DCS 4 course (N = 32)
Age (years)	Median (range)	65 (36‐79)	61 (27‐75)	64 (30‐80)	66.5 (37‐80)
Gender	Male	18 (58%)	17 (55%)	23 (70%)	18 (56%)
	Female	13 (42%)	14 (45%)	10 (30%)	14 (44%)
Performance status	0	27 (87%)	26 (84%)	31 (94%)	28 (88%)
	1	4 (13%)	5 (16%)	2 (6%)	4 (12%)
Macroscopic type	Type 1	0 (0%)	1 (3%)	2 (6%)	2 (6%)
	Type 2	5 (16%)	7 (23%)	5 (15%)	7 (22%)
	Type 3	20 (65%)	19 (61%)	14 (42%)	16 (50%)
	Type 4	6 (19%)	3 (10%)	11 (33%)	7 (22%)
	Others	0 (0%)	1 (3%)	1 (3%)	0 (0%)
Histological type	Differentiated	10 (32%)	13 (42%)	11 (33%)	15 (47%)
	Undifferentiated	21 (68%)	18 (58%)	22 (67%)	17 (53%)
Location	GEJ*	8 (26%)	10 (32%)	8 (24%)	8 (25%)
	Stomach	23 (74%)	21 (68%)	25 (76%)	24 (75%)
Clinical T factor	T3	4 (13%)	4 (13%)	4 (12%)	7 (22%)
	T4a	23 (74%)	25 (81%)	26 (79%)	23 (72%)
	T4b	4 (13%)	2 (6%)	3 (9%)	2 (6%)
Clinical T factor	N0	8 (26%)	7 (23%)	11 (33%)	8 (25%)
	N+	23 (74%)	24 (77%)	22 (67%)	24 (75%)

### 3.2 Prognosis

**FIGURE 2:**
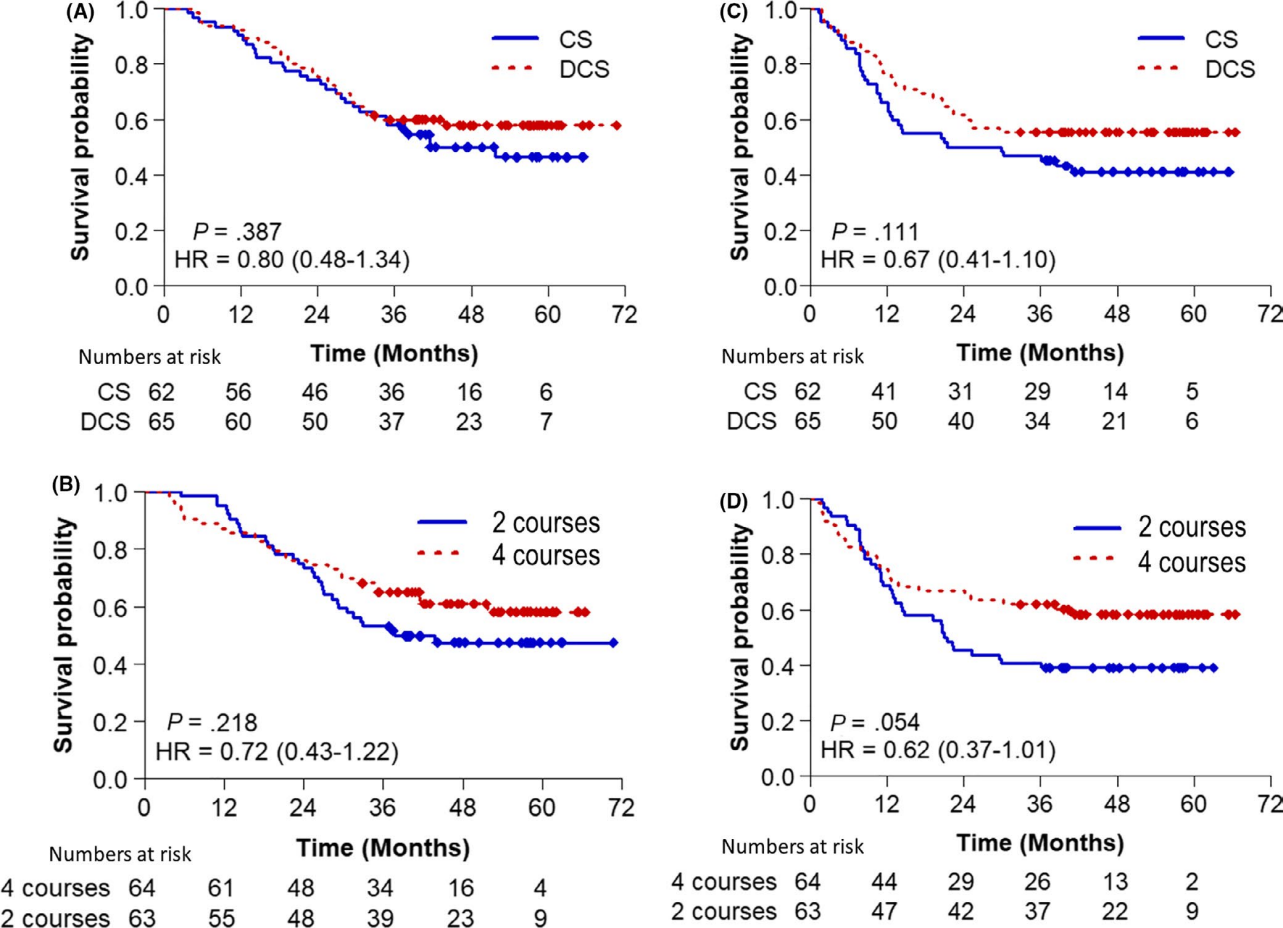
Overall survival by regimen (A) and by courses (C). Progression‐free survival by regimen (B) and by courses (D)

The median follow‐up was 47.6 months for arm A, 51.5 months for arm B, 50.5 months for arm C, and 53.8 months for arm D. Figure 2 shows OS curves stratified by regimens (Figure 2A), number of courses (Figure 2B), and PFS (Figure 2C,D). The 3‐year OS rate was 58% (95% CI 46%‐70%) in the CS, 60% (95% CI 48%‐72%) in the DCS, 53% (95% CI 41%‐65%) in two courses, and 65% (95% CI 53%‐77%) in four courses. There was no significant difference in both regimens and number of courses.

The hazard ratio for death in the DCS compared to CS was 0.80 (95% CI 0.48‐1.34), and it was 0.72 (95 CI 0.43‐1.22) in four courses compared to two courses.

The 3‐year PFS rate was 47% (95% CI 34%‐59%) in the CS, 55% (95% CI 43%‐68%) in the DCS, 41% (95% CI 29%‐53%) in two courses, and 62% (95% CI 50%‐74%) in four courses. No statistically significant differences were observed between the regimen and number of courses. The HR for PFS in the DCS groups compared to the CS groups was 0.67 (95% CI 0.41‐1.10), while it was 0.62 (95% CI 0.37‐1.01) in the four‐course groups compared to the two‐course groups.

**FIGURE 3:**
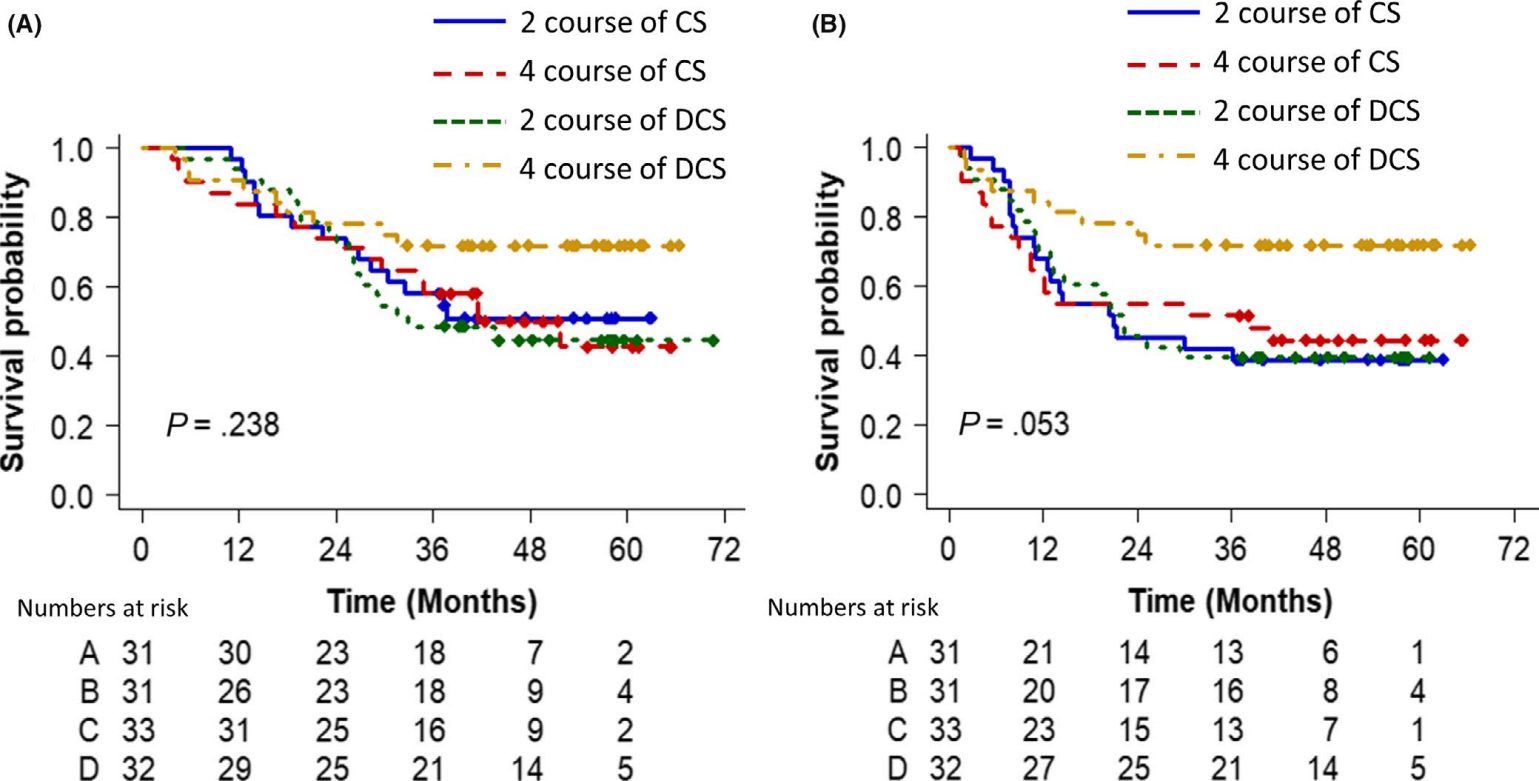
Overall survival of each arm (A). Progression‐free survival of each arm (B)

The OS curves for all the eligible patients in each arm are shown in Figure 3A, and the PFS in Figure 3B. The 3‐year OS rate was 58% (95% CI 41%‐75%) in arm A, 58% (95% CI 41%‐75%) in arm B, 49% (95% CI 31%‐66%) in arm C, and 72% (95% CI 56%‐86%) in arm D. Although no significant differences were observed among these four arms (*P* = .2377), the 3y‐OS of four courses DCS (arm D) was 14% better than the that of two courses CS (arm A) as a reference arm. The hazard ratio for death in comparison to the arm A was 1.06 (95% CI 0.53‐2.15) in arm B, 1.15 (95% CI 0.58‐2.28) in arm C, and 0.52 (95% CI 0.23‐1.20) in arm D.

The 3‐year PFS rate was 42% (95% CI 25%‐59%) in arm A, 52% (95% CI 34%‐69%) in arm B, 39% (95% CI 23%‐56%) in arm C, and 72% (95% CI 56%‐88%) in arm D. Although the PFS was better in arm D than in the other arms, there was no significant difference among these four arms (*P* = .053). The HR for PFS in comparison to arm A was 0.91 (95% CI 0.47‐1.76) in arm B, 0.96 (95% CI 0.51‐1.79) in arm C, and 0.38 (95% CI 0.17‐0.84) in arm D.

**FIGURE 4:**
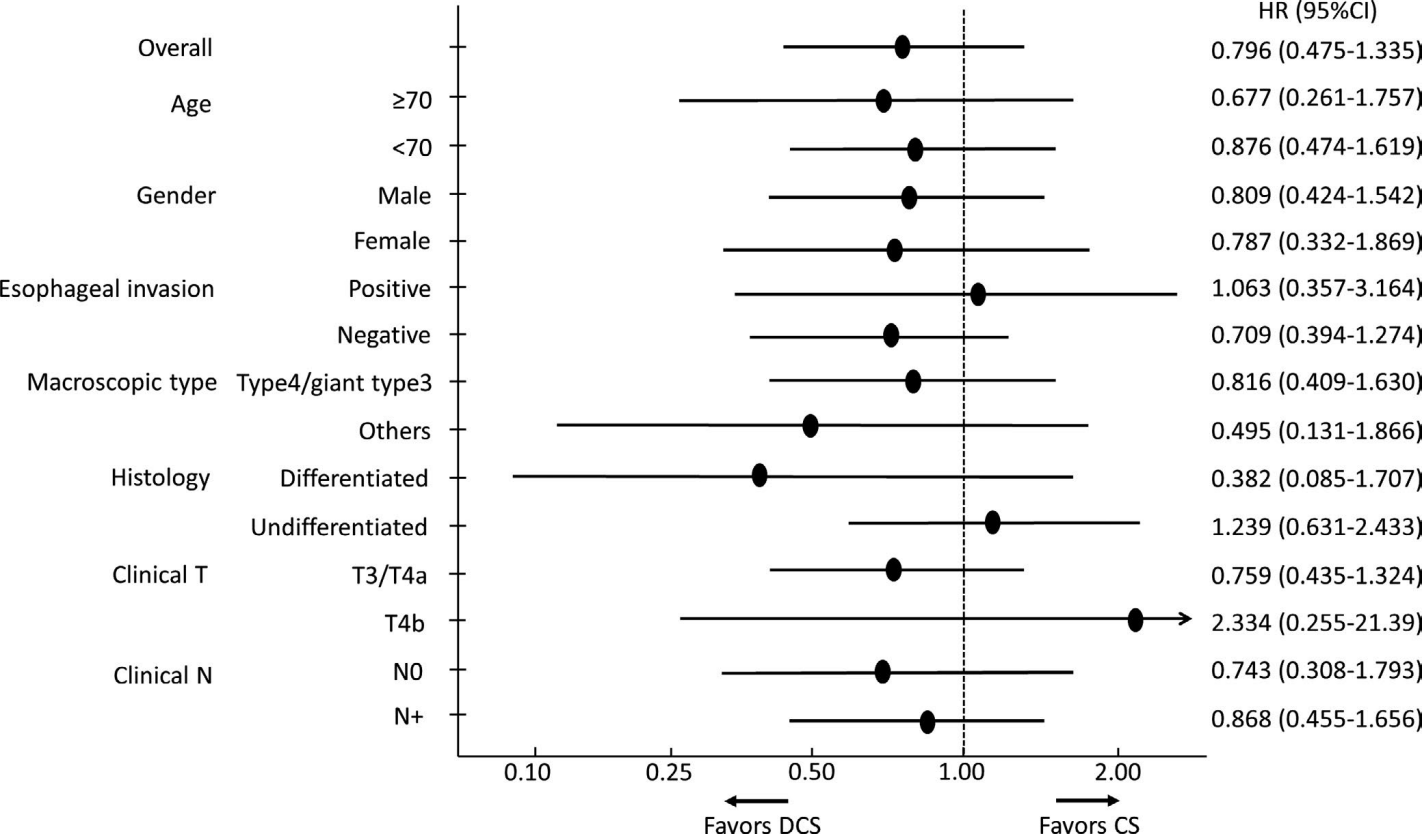
Forest plot showing of the treatment effects for the overall survival by regimen

**FIGURE 5:**
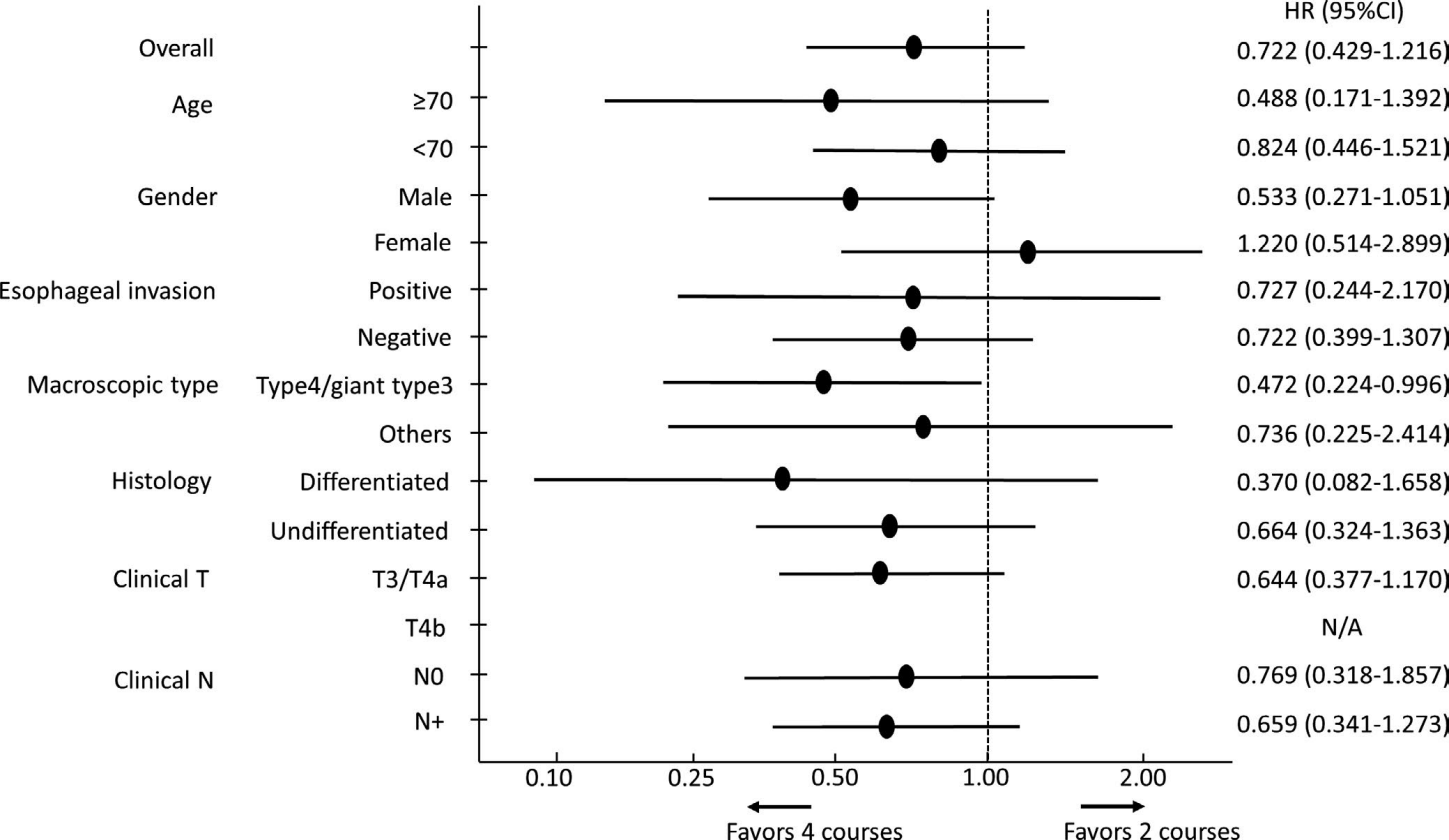
Forest plot showing the treatment effects for the progression‐free survival by courses

The forest plots presenting effects of treatment on OS are shown in Figure 4 by regimen and in Figure 5 by number of courses. Remarkably, four courses were better in type 4 and giant type 3 than in other macroscopic types.

## 4 DISCUSSIONS

The major finding of the present trial was that the four‐course neoadjuvant chemotherapy achieved a 10% improvement in 3‐year OS compared with the two courses of chemotherapy, which met the primary endpoint of present trial. Additionally, 3‐year OS and PFS rate of four courses of DCS regimen was the highest among the arms. This suggestion is made that four courses of DCS regimen of neoadjuvant chemotherapy is recommended as a promising arm in a future phase III study for locally advanced gastric cancer.

Efficacy of docetaxel has been confirmed in two phase III trials of adjuvant chemotherapy. The first one is the FLOT‐AIO trial,^7^ conducted in Germany, which demonstrated that overall survival was better in patients who received perioperative chemotherapy with the docetaxel/oxaliplatin/leucovorin/fluorouracil (FLOT) than those treated with epirubicin/cisplatin/fluorouracil or capecitabine (ECF/ECX). The other one is the START2 trial,^4^ conducted in Japan. The START2 showed that postoperative S‐1 plus docetaxel had superior survival as compared with S‐1 for stage III. Hazard ratio (HR) was 0.77 in the former and 0.632 in the latter. In the present study, HR of DCS to CS was almost concordant to the FLOT study.^7^ The present study has several similarities to the FLOT study; control was platinum containing regimen and neoadjuvant setting. It is interesting that similar HR was confirmed in the present study, overcoming the difference of race, location, and surgery between Germany and Japan. Meanwhile, the triplet regimen with docetaxel, S‐1, and cisplatin failed to demonstrate superiority to the doublet regimen with S‐1 and cisplatin for the patients with unresectable or recurrent gastric cancer in the JCOG1013 study,^17^ which was inconsistent with the results of the present study. This discrepancy might be explained by the difference between palliative and neoadjuvant settings. Another important trial^18^ is the Korean PRODIGY study which confirms efficacy of additional neoadjuvant chemotherapy containing S‐1, oxaliplatin, and docetaxel against the control of surgery followed by adjuvant S‐1 alone. That trial demonstrated efficacy of neoadjuvant chemotherapy in progression‐free survival, however, did not show any benefit in the overall survival.

Another interesting result was that four‐course arm had superior survival than two‐course arm, which was apparent only in DCS regimen. Previously, two phase III trials comparing duration of chemotherapy for locally advanced gastric cancer showed that no survival benefit was obtained by longer period of adjuvant chemotherapy. The first trial^19^ had compared mitomycin C and short doxifluridine for 6 months with mitomycin C, long doxifluridine, and cisplatin for 12 months. Another trial^20^ had compared two courses of fluorouracil plus cisplatin with four courses of capecitabine, cisplatin, and epirubicin. Recent international IDEA collaboration for colon cancer also showed that shortening the duration of adjuvant chemotherapy using FOLFOX or capecitabine plus oxaliplatin did not worsen the survival.^21^ In our previous phase II study, two and four courses of CS regimen and S‐1 plus paclitaxel had similar survival rates. The case for CS regimen was confirmed in the present study. So far, there was no study to compare duration of docetaxel in the adjuvant or neoadjuvant setting. Previously, we clarified that the rate of pathological response,^16^ defined by less than 10% of the residual tumor remaining, was 12.1% in two courses of DCS and 18.8% in four courses of DCS. The better survival could be explained by an increase of pathological response. On the other hand, we had reported that the pathological response was both 19.4% in the two and four courses CS, which will explain the present result that the survival was similar between two and four courses of CS.

In the present study, the pathological response of DCS was slightly lower than the CS regimen. Thus, the better survival by DCS found in the present study was contradictory to the pathological response. Although exact mechanisms are unclear, one possibility would be diagnostic accuracy of pathological response. As background factors, frequency of type 4 or scirrhous type was 27.7% (18/65) in the DCS arm while it was 14.5% (9/62) in the CS arm. Type 4 gastric cancer is characterized by diffuse infiltration of the tumor cells together with proliferation of collagen tissue in the thickened wall of the stomach, by which it would be difficult to identify the residual tumor area and the area occupied by the primary tumor. Previously, Nakamura^22^ reported that the pathological response did not predict the survival in type 4 disease. Although exact mechanisms are unclear, one possibility might be diagnostic accuracy of pathological response.

Recently, JCOG0501 study,^23^ conducted in Japan, failed to demonstrate the survival benefit for preoperative administration of two courses of CS as a NAC setting in addition to postoperative S‐1 adjuvant chemotherapy for locally advanced Type 4 or large Type 3 gastric cancer. Although CS of NAC for the common type was unclear, more effective regimen than CS must be developed. In the subgroup analysis of the present study, four‐course regimen had tended to achieve better survival than two‐course regimen both in type 4/giant type 3 and the other macroscopic types. Similar tendency was also observed in the case for DCS regimen as compared with the case for CS regimen. Our results would suggest that type 4/giant type 3 and the other macroscopic types would not be separately treated when conducting phase III study to show the efficacy of neoadjuvant chemotherapy of four‐course regimen or DCS regimen.

Generally, 5‐year OS or 3‐year RFS is a suitable primary endpoint to evaluate the efficacy of adjuvant chemotherapy in the phase III trial. Different from adjuvant chemotherapy, however, event for RFS in the case of neoadjuvant chemotherapy can not reduce a little uncertainly because it would be unclear whether peritoneal metastasis found at the surgery is due to progression or limitation of CT diagnosis at the enrollment. Thus, RFS has not been established as a surrogate endpoint in case of neoadjuvant chemotherapy. Moreover, a shorter follow‐up period is more favorable because our aim is not a definitive result but is to explore a better regimen and course for future phase III study. Based on these, we set 3‐year OS as the primary endpoint in this phase II study.

In conclusion, four‐course DCS regimen of neoadjuvant chemotherapy is recommended as a promising regimen in a future phase III study for locally advanced gastric cancer.

## DISCLOSURE

Conflict of Interest: The authors have declared no conflicts of interest.

Funding: This work was supported, in part, by the nongovernmental organization Kanagawa Standard Anti‐cancer Therapy Support System (no grant numbers apply).
